# Regulatory mechanisms of hypoxia-inducible factors on ferroptosis in ischemic stroke

**DOI:** 10.7717/peerj.21274

**Published:** 2026-06-03

**Authors:** Shengshan Yuan, Phaik Har Yong, Zhi Xiang Ng, Lijian Wei, Baoyan Ren, Lina Liang, Guijiang Wei

**Affiliations:** 1 Department of Neurology, Affiliated Hospital of Youjiang Medical University for Nationalities, Baise, Guangxi, China; 2 School of Bioscience, Faculty of Pharmacy and Biomedical Sciences, MAHSA University, Selangor, Malaysia; 3 School of Biological and Environmental Sciences, Faculty of Science and Engineering, University of Nottingham Malaysia, Selangor, Malaysia; 4 Center for Medical Laboratory Science, Affiliated Hospital of Youjiang Medical University for Nationalities, Baise, Guangxi, China; 5 Key Laboratory of Research on Clinical Molecular Diagnosis for High Incidence Diseases in Western Guangxi of Guangxi Higher Education Institutions, Baise, Guangxi, China; 6 Engineering Research Center of Guangxi Higher Education Institutions for Precise Genetic Testing of Long-dwelling Nationalities, Baise, Guangxi, China; 7 Guangxi Engineering Research Center for Precise Genetic Testing of Long-dwelling Nationalities, Baise, Guangxi, China; 8 Yaneng BlOscience (Shenzhen) Corporation, Shenzhen, Guangdong, China

**Keywords:** Ischemic stroke, HIF-1α, HIF-3α, Ferroptosis, Pathway

## Abstract

Ischemic stroke remains a primary contributor to global disability and mortality, primarily resulting from interruptions in cerebral blood flow that trigger brain tissue necrosis. Among the various mechanisms of cell death, ferroptosis assumes a key role in the pathophysiology of cerebral infarction. Hypoxia-inducible factors (HIFs) are central to cellular adaptation under hypoxic conditions and exhibits dual regulatory roles in neuronal ferroptosis. This review delineates the intricate mechanisms by which HIF-1α and the less-characterized HIF-3α modulate ferroptosis in ischemic stroke. We detail how HIF-1α promotes ferroptosis by disrupting iron homeostasis, amplifying lipid peroxidation, and activating inflammatory networks, while also highlighting its context-dependent protective functions. Furthermore, we explore the emerging role of HIF-3α, particularly its mitochondrial localization and diverse functions conferred by alternative splicing variants, which may oppose HIF-1α-driven ferroptosis. By synthesizing the distinct regulatory networks of HIF-1α and HIF-3α, this review underscores their potential as therapeutic targets and proposes that selective modulation of these pathways could offer novel neuroprotective strategies for ischemic stroke.

## Introduction

Ischemic stroke induces neuronal death and subsequent neurological deficits due to cerebral blood flow interruption, primarily caused by vascular occlusion through thrombosis, embolism, or systemic hypoperfusion (Campbell et al., 2019; Smith et al., 2018). According to the most recent Global Burden of Disease (GBD) analyses, stroke remains a major global health challenge, accounting for more than 12 million incident cases annually. In addition, stroke representing the second leading cause of death, and a leading cause of long-term disability worldwide (GBD 2021 Stroke Risk Factor Collaborators, 2024; Wang et al., 2022; Yeh et al., 2023). Ischemic stroke constitutes approximately 60–70% of all stroke cases, thereby contributing substantially to global mortality and disability adjusted life years (Hilkens et al., 2024; Li et al., 2025).

Ischemic stroke initiates a cascade of biochemical processes culminating in cellular death in the brain. Major forms of cell death observed post-stroke include necrosis, apoptosis, and the recently identified ferroptosis (Chen & Jin, 2023; Li et al., 2024a; Zhu et al., 2024a). Ferroptosis is a cell death modality closely associated with dysregulated iron metabolism and lipid peroxidation (Chen et al., 2021a; Co et al., 2024; Stockwell et al., 2017; Stockwell, 2022; Zhang et al., 2021a), which disrupts cellular membrane integrity and initiates programmed cell death (Hirschhorn & Stockwell, 2019). Neurons, with their high oxidative metabolic demands and iron-rich microenvironment, are particularly susceptible to ferroptosis (Li et al., 2024a; Ryan et al., 2023). Its role in neurodegenerative diseases and cerebral ischemic injury has attracted increasing attention in recent years (Hirschhorn & Stockwell, 2019; Li et al., 2020a; Li et al., 2024a; Liu et al., 2024). As a critical pathological mechanism in neurological disorders such as ischemic stroke, ferroptosis contributes significantly to post-stroke neuronal damage (Chen et al., 2021b; Kang, Kroemer & Tang, 2019; Li et al., 2021; Tuo et al., 2022). Inhibiting key processes in ferroptosis, such as lipid peroxidation and iron accumulation, has demonstrated protective impacts against ischemic injury (Zhao et al., 2022), suggesting that therapeutic targeting of ferroptosis may offer an effective strategy to mitigate stroke severity and enhance recovery.

**Figure 1:**
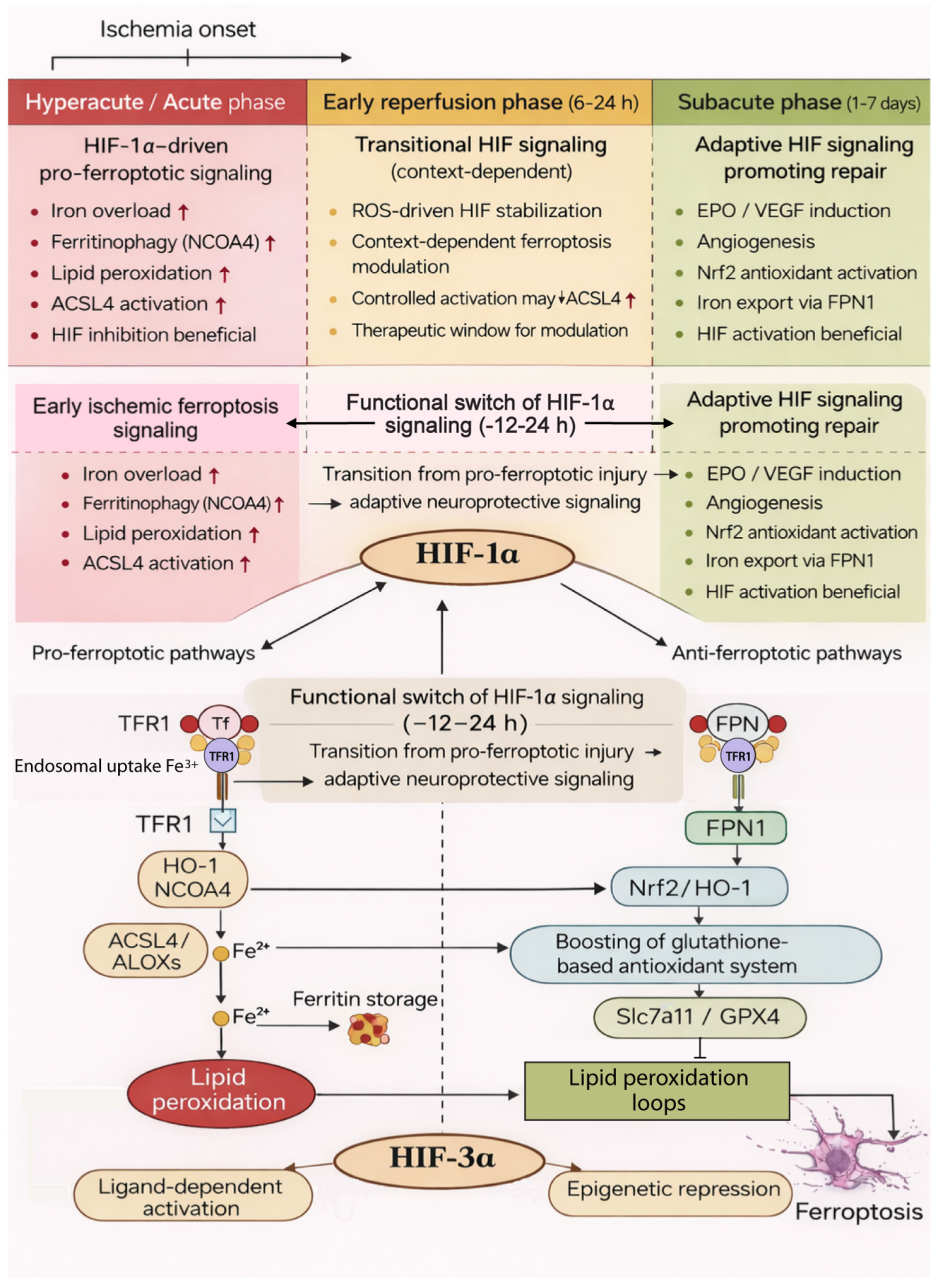
Schematic diagram of the molecular networks by which HIF-1α and HIF-3α regulate ferroptosis in ischemic stroke. Under hypoxic conditions, HIF-1α exerts bidirectional effects. Its pro-ferroptotic pathways (left) include upregulating iron import (TfR1) and release (HO-1, BNIP3/NCOA4), inhibiting iron storage (FTH1) and export (FPN1), amplifying lipid peroxidation (ACSL4, ALOXs), and activating inflammatory feedback loops (TNF-α/IL-6/NF-κ B/NOX4). Conversely, its anti-ferroptotic pathways (right) involve enhancing iron efflux (FPN1), activating the Nrf2/HO-1 axis, and boosting the glutathione-based antioxidant system (Slc7a11, GPX4). HIF-3α modulates ferroptosis through dominant-negative regulation of HIF-1α, mitochondrial function regulation, epigenetic repression (HIF-3α4/HDAC1), and ligand-dependent activation. Solid arrows indicate activation; blunt arrows indicate inhibition.

Hypoxia-inducible factors (HIFs) are heterodimeric transcription factors composed of α and β subunits, with the α subunit including HIF-1α, HIF-2α, and HIF-3α (Li et al., 2020b). These transcription factors sense cellular oxygen levels and regulate the expression of oxygen responsive genes (Zhang et al., 2014). Under hypoxic stress, they play pivotal roles in maintaining cell survival by modulating genes involved in angiogenesis, hematopoiesis, glucose utilization, iron metabolism, extracellular matrix synthesis, cell proliferation, and apoptosis, which are essential for cellular adaptation to low oxygen environments (Amalia et al., 2020; Jaskiewicz et al., 2022; Pasanen et al., 2010; Wicks & Semenza, 2022; Zhang et al., 2022). While this review focuses on the distinct roles of HIF-1α and HIF-3α, it is important to note that HIF-2α also contributes to hypoxia adaptation, particularly in regulating erythropoietin and certain iron metabolism genes (Chu et al., 2025; Płoszczyca et al., 2023; Zhu et al., 2020). However, its role in neuronal ferroptosis remains less defined compared to HIF-1α and warrants further investigation. Their dual regulatory roles in ferroptosis have recently garnered increasing attention. This review concentrates on elucidating the molecular networks of HIF-1α and HIF-3α in neuronal ferroptosis, and constructs a core signaling network diagram of HIF-mediated ferroptosis (Fig. 1) and illustrates its pathological roles and therapeutic targets in ischemic stroke by reviewing relevant mechanisms. A deeper understanding of HIF-mediated ferroptosis mechanisms will provide valuable insights into ischemic stroke pathogenesis and inform novel therapeutic strategies.

## Why Is This Review Needed, and Who Is It Intended For?

Ferroptosis is a key molecular mechanism in ischemic stroke. In recent years, the regulatory role of HIFs in ferroptosis has garnered considerable interest. However, a comprehensive review specifically addressing the mechanisms and therapeutic potential of HIF-1α and HIF-3α in regulating ferroptosis during cerebral infarction is currently lacking. This review systematically elaborates the molecular mechanisms through which HIF-1α and HIF-3α modulate ferroptosis, their pathological roles, and their potential therapeutic applications in ischemic stroke. Our review will appeal to neuroscientists and other researchers interested in hypoxia-inducible factors and ferroptosis, while providing them with novel innovative concepts and insights for future experimental approaches in the treatment of cerebral infarction.

## Search Strategy

We conducted a systematic literature search using PubMed and Web of Science, with the final search updated to October 10, 2025. The search was restricted to full-text journal articles and focused on the ferroptosis pathway regulated by HIF-1α and HIF-3α, along with its role in cerebral infarction. Keywords were grouped by category using a synonym and variant classification approach, including:

1. Ischemic stroke: cerebral infarction, cerebral ischemia-reperfusion, acute cerebral infarction.

2. Ferroptosis: ferroptosis, anti-ferroptosis, mechanism of ferroptosis, lipid peroxidation.

3. Hypoxia-inducible factors: HIF-1α, HIF-3α, HIFs.

Boolean operators (AND/OR) were applied to combine search terms. The initial search yielded approximately 510 potentially relevant articles for this review.

### Molecular mechanisms of HIF in regulating ferroptosis

The HIF family, particularly HIF-1α, serves as a key molecular regulator of ferroptosis, primarily promoting this process by driving iron overload, lipid peroxidation, and inflammatory networks. However, it also exhibits protective anti-ferroptotic functions under specific contexts (Yao et al., 2024). HIF-3α possesses multiple splice variants that participate in regulating various physiological and pathological processes under both hypoxic and normoxic conditions. Through these intricate regulatory networks, HIF-1α and HIF-3α play central roles in hypoxia induced ferroptosis in neural cells.

### HIF-2α and its long-term role in hypoxic adaptation and recovery

Although hypoxia-inducible factors collectively coordinate cellular adaptation to reduced oxygen availability, their temporal dynamics and functional specificity differ substantially. HIF-2α is increasingly recognized as a key regulator of sustained hypoxic adaptation, particularly under moderate and prolonged hypoxia (Cheng et al., 2024). In contrast to the rapid and transient activation of HIF-1α during acute oxygen deprivation, HIF-2α demonstrates a more delayed stabilization profile and is preferentially maintained during chronic hypoxic exposure (Bakleh & Al Haj Zen, 2025). These characteristics position HIF-2α as an important mediator of long-term vascular remodeling, metabolic reprogramming, and tissue repair rather than immediate injury responses.

Functionally, HIF-2α regulates genes involved in angiogenesis, mitochondrial metabolism, and endothelial homeostasis (Cheng et al., 2024). It exerts comparatively weaker control over glycolytic enzymes but plays a more prominent role in lipid metabolism and oxidative phosphorylation pathways (Bakleh & Al Haj Zen, 2025), supporting sustained cellular adaptation rather than rapid metabolic switching. Furthermore, HIF-2α contributes to endothelial barrier integrity, angiogenesis, and cytoprotective signaling during ischemia-reperfusion injury (Huang & Zhao, 2025), reinforcing its relevance in recovery and remodeling phases.

Taken together, these findings suggest that while HIF-2α is critically involved in long-term hypoxic adaptation and post-injury repair, the immediate and dominant transcriptional response to acute ischemia is primarily mediated by HIF-1α. Therefore, given the central importance of early hypoxic signaling in the pathophysiology of ischemic injury, this review focuses predominantly on HIF-1α, while acknowledging the complementary and recovery associated roles of HIF-2α.

### Bidirectional regulatory role of HIF-1α

HIF-1α is a central mediator of cellular responses to hypoxia, controlling the expression of numerous target genes that are involved in both adaptive and pathological processes (Mitroshina et al., 2021; Wicks & Semenza, 2022). Its activation is directly triggered by reduced intracellular oxygen tension, with enhanced stability and transcriptional activity serving as adaptive mechanisms under hypoxic conditions. HIF-1α is closely linked to ferroptosis by modulating the transcription of various iron metabolism- and ferroptosis-associated genes (Xu, Wang & Xie, 2017), which establishes a complex, context-dependent regulatory paradigm.

### Pro-ferroptotic mechanisms of HIF-1α: normoxic suppression *versus* hypoxic activation

Under normoxic conditions, HIF-1α is continuously synthesized but rapidly hydroxylated by prolyl hydroxylase domain enzymes, allowing recognition by the von Hippel-Lindau (VHL) E3 ubiquitin ligase complex and subsequent proteasomal degradation. This tight oxygen-dependent regulation prevents transcriptional activation of hypoxia-responsive genes and preserves metabolic homeostasis. Consequently, canonical HIF-1α downstream targets involved in glycolysis, mitochondrial remodeling, and stress adaptation remain minimally expressed (Lee, Chandel & Simon, 2020). In this state, pyruvate dehydrogenase activity is maintained, oxidative phosphorylation predominates, and mitochondrial respiration proceeds efficiently (Lee, Chandel & Simon, 2020). As a result, iron-handling, lipid remodeling, and redox-altering gene programs that may predispose cells to ferroptosis are not transcriptionally amplified.

In contrast, hypoxic stabilization of HIF-1α initiates a broad transcriptional program *via* hypoxia response elements. Representative downstream targets include glycolytic and metabolic regulators such as solute carrier family 2 member 1 (SLC2A1; GLUT1), solute carrier family 2 member 3 (SLC2A3; GLUT3), hexokinase 2 (HK2), pyruvate kinase M2 (PKM2), lactate dehydrogenase A (LDHA), and pyruvate dehydrogenase kinase 1 (PDK1), mitochondrial modulators BCL2/adenovirus E1B 19 kDa interacting protein 3 (BNIP3) and BCL2/adenovirus E1B 19 kDa interacting protein 3-like (BNIP3L), and glutamine transporters solute carrier family 1 member 5 (SLC1A5) and solute carrier family 38 member 2 (SLC38A2) (Lee, Chandel & Simon, 2020). Through coordinated regulation of these genes, HIF-1α orchestrates metabolic reprogramming, mitochondrial remodeling, and redox adaptation. Overall, these transcriptional shifts reshape intracellular iron utilization, lipid biosynthesis, and reactive oxygen species (ROS) dynamics, thereby establishing a mechanistic framework through which HIF-1α can influence ferroptotic susceptibility under hypoxic stress.

The pro-ferroptotic effects of HIF-1α under hypoxia can be conceptualized across four interrelated mechanistic dimensions.

### Metabolic reprogramming and redox imbalance

HIF-1α strongly induces glycolytic genes, including GLUT1, GLUT3, HK1/2, PKM2, and LDHA, thereby promoting glucose uptake and lactate production (Lee, Chandel & Simon, 2020). Simultaneously, upregulation of PDK1 inhibits pyruvate dehydrogenase, suppressing pyruvate entry into the tricarboxylic acid cycle and limiting mitochondrial oxidative phosphorylation (Lee, Chandel & Simon, 2020).

This metabolic shift toward aerobic glycolysis alters NADH/NAD^+^ balance and reduces mitochondrial respiration efficiency, thereby reshaping intracellular redox homeostasis. Hypoxia also modulates mitochondrial ROS signaling, which contributes to HIF-1α stabilization and downstream adaptive responses (Lee, Chandel & Simon, 2020). While these adaptations initially serve protective roles, persistent redox imbalance under sustained hypoxia may sensitize cells to lipid peroxidation, a defining event in ferroptosis.

### Iron homeostasis dysregulation

Beyond metabolic reprogramming, HIF-1α exerts profound control over iron metabolism, a central determinant of ferroptotic execution. HIF-1α upregulates transferrin receptor 1 (TfR1) by binding to hypoxia response elements in its promoter region, thereby enhancing cellular iron uptake (Semenza, 2001). Concurrently, HIF-1α has been reported to suppress ferritin heavy chain (FTH1), increasing the intracellular labile Fe^2^^+^ pool and intensifying Fenton chemistry (Dixon et al., 2012; Liu, Kang & Tang, 2022).

In addition, HIF-1α induces heme oxygenase-1 (HO-1), which catalyzes heme degradation and releases free iron, further contributing to iron accumulation. HIF-1α also regulates microRNA-210, which has been implicated in maintaining TfR expression and modulating iron homeostasis (Chan et al., 2009; Yoshioka et al., 2012).

Collectively, these regulatory events elevate intracellular iron availability, thereby facilitating iron-dependent lipid peroxidation and ferroptotic vulnerability.

### Lipid metabolism rewiring and amplification of lipid peroxidation

Ferroptosis is critically dependent on the accumulation of oxidized phospholipids containing polyunsaturated fatty acids (PLs-PUFA). HIF-1α-mediated metabolic reprogramming enhances carbon flux toward lipid biosynthesis by sustaining cytosolic acetyl-CoA production through glycolytic and glutamine-dependent pathways (Lee, Chandel & Simon, 2020).

In certain contexts, HIF-1α has been associated with upregulation of acyl-CoA synthetase long-chain family member 4 (ACSL4), which facilitates the incorporation of polyunsaturated fatty acids into membrane phospholipids (Cui et al., 2021; Doll et al., 2017). Lipoxygenases (ALOXs) subsequently oxidize PLs-PUFA, generating lipid peroxides that destabilize membrane integrity and drive ferroptotic cell death (Minotti & Aust, 1989; Rochette et al., 2022).

Furthermore, HIF-1α may downregulate key ferroptosis suppressors such as SLC7A11 and ferroptosis suppressor protein 1 (FSP1) in specific cellular contexts (Conrad et al., 2016), thereby weakening antioxidant defenses and amplifying lipid peroxidation cascades.

### Stress signaling and inflammatory crosstalk

Hypoxia-induced HIF-1α activation intersects with multiple stress and inflammatory pathways that further influence ferroptotic outcomes (Chen et al., 2023). Activation of unfolded protein response branches, including the protein kinase RNA-like endoplasmic reticulum kinase (PERK)-eukaryotic initiation factor 2 alpha (eIF2α), and inositol-requiring enzyme 1 alpha (IRE1α)-X-box binding protein 1 (XBP1) signaling pathways (Lee, Chandel & Simon, 2020), integrates endoplasmic reticulum stress with hypoxic adaptation. XBP1 can interact with HIF-1α to enhance transcription of hypoxia-responsive genes, amplifying stress-adaptive networks.

In ischemia-reperfusion settings, inflammatory cytokines such as TNF-α and IL-6 may activate nuclear factor-κB (NF-κB) signaling, which can further promote HIF-1α expression and establish positive feedback loops linking hypoxia, inflammation, and oxidative stress (Bell, Stockwell & Zou, 2024). ROS generated within inflammatory microenvironments inhibit prolyl hydroxylases, stabilizing HIF-1α. HIF-1α may, in turn, enhance oxidative stress by upregulating ROS-generating enzymes such as NOX4 (Wu, He & Zhu, 2018; Yang et al., 2022), thereby reinforcing lipid peroxidation and ferroptotic signaling.

### Anti-ferroptotic mechanisms of HIF-1α

Although HIF-1α is typically associated with promoting ferroptosis, emerging evidence has suggested its potential protective roles against ischemic necrosis. Under certain conditions, modulating HIF-1α activity can reduce ferroptosis and protect neural cells from ischemic damage. For example, HIF-1α could suppress ferroptosis in cerebral neurons and ameliorate ischemia-reperfusion injury by inducing ferroportin 1 expression and activating the nuclear factor erythroid-related factor 2 (Nrf2)/ HO-1 pathway (Guo et al., 2024; Hu et al., 2022; Yao et al., 2024). HIF-1α upregulation enhances the expression of Slc7a11 and glutathione peroxidase 4 (GPX4), increasing cysteine and glutathione levels while suppressing ROS accumulation, thereby promoting cellular damage repair (An et al., 2024). HIF-1α also boosts the transcription of the glutamate transporter SLC1A1 and facilitates cystine uptake to confer resistance to ferroptosis (Yang et al., 2023).

### The unique regulatory network of HIF-3α in ferroptosis

As one of the critical members of the HIF family, HIF-3α has collected significant attention in recent years. The human HIF-3α gene generates multiple splice variants through alternative promoters, transcription start sites, and splicing. These variants exhibit tissue and developmental stage-specific expression, dynamically regulated by hypoxia and other factors (Duan, 2016; Pasanen et al., 2010). Due to the lack of an oxygen-dependent degradation domain in some variants, HIF-3α can remain stably expressed under normoxic conditions, contributing to its functional complexity (Duan, 2016).

HIF-3α participates in ferroptosis regulation through several unique mechanisms, as detailed in Table 1. Several HIF-3α variants can act as dominant-negative regulators of HIF-1α/2α by competing for the shared HIF-β subunit (Suzuki et al., 2017). Furthermore, studies indicate that mitochondrial-localized HIF-3α modulates ferroptosis through epigenetic regulation and endogenous ligand-mediated mechanisms. For example, HIF-3α anchored to the mitochondrial outer membrane can regulate mitochondrial ROS production and iron-sulfur cluster biosynthesis, thereby influencing ferroptosis susceptibility (Li et al., 2019). The HIF-3α4 variant recruits histone deacetylase 1 (HDAC1) to repress HIF-1α target genes, such as vascular endothelial growth factor (VEGF), thereby indirectly modulating iron metabolism homeostasis (Ando et al., 2013). Additionally, oleoylethanolamide (OEA), a specific ligand of HIF-3α, enhances the stability of the HIF-3α- aryl hydrocarbon receptor nuclear translocator heterodimer and boosts HIF-3α transcriptional activity, potentially promoting antioxidant gene expression (Diao et al., 2022).

**Table 1:** Molecular mechanisms and pathways of HIF in regulating ferroptosis.

**Category**	**Molecular mechanism**	**Pathway/target**	**Functional effect**	**Key references**
HIF-1α Pro-Ferroptosis Mechanisms	Iron metabolism dysregulation	↑HO-1, ↑TfR1, ↓FTH1	Catalyzes heme degradation to release free iron; enhances cellular iron uptake; inhibits iron storage → Promotes Fenton reaction	Guo et al., 2024; Li et al., 2021
	Ferritinophagy activation	↑BNIP3/NCOA4	Promotes ferritin degradation and free iron release → Increases intracellular iron load	Mitroshina et al., 2021
	Iron efflux suppression	↓Ferroportin1, ↑miR-210	Inhibits ferroportin expression; impairs mitochondrial iron utilization → Cytosolic iron accumulation	Chan et al., 2009; Yoshioka et al., 2012
	Lipid peroxidation amplification	↑ACSL4, ↑ALOXs	Promotes oxidation of polyunsaturated fatty acids (PUFAs) → Lipid peroxide accumulation, membrane damage	Doll et al., 2017; Tuo et al., 2022
	Inflammatory signaling network	TNF-α/IL-6 → NF-κB →↑HIF-1α	Forms a positive feedback loop; upregulates ROS-generating enzymes (*e.g.,* NOX4) → Amplifies oxidative stress	Bell, Stockwell & Zou, 2024; Yang et al., 2022
HIF-1α Anti-Ferroptosis Mechanisms	Iron homeostasis protection	↑Ferroportin1, Activation of Nrf2/HO-1 pathway	Enhances iron efflux, alleviates intracellular iron overload → Inhibits lipid peroxidation	Yao et al., 2024
	Antioxidant defense	↑Slc7a11/GPX4, ↑SLC1A1	Increases cystine uptake and glutathione synthesis → Enhances ROS scavenging capacity	An et al., 2024; Yang et al., 2023
HIF-3α Unique Regulatory Network	Dominant-negative regulation	Competes for HIF-β subunit	Inhibits HIF-1α/2α transcriptional activity → Indirectly regulates iron metabolism homeostasis	Suzuki et al., 2017
	Mitochondrial function regulation	↓Mitochondrial ROS, ↑Iron-sulfur cluster biosynthesis	Maintains mitochondrial redox balance → Reduces ferroptosis susceptibility	Jiang et al., 2024; Li et al., 2019
	Epigenetic regulation	HIF-3α4-HDAC1 complex →↓VEGF	Suppresses pro-ferroptosis gene expression	Ando et al., 2013
	Ligand-dependent activation	Oleoylethanolamide (OEA) binding	Stabilizes HIF-3α-ARNT dimer → Enhances transcription of antioxidant genes	Diao et al., 2022

Despite these intriguing findings, the functional consequences of specific HIF-3α splice variants in the ischemic brain are not yet fully elucidated. Future studies employing isoform-specific knockdown or overexpression in animal models are crucial to determine whether HIF-3α primarily serves as a context-dependent antagonist of HIF-1α/2α or possesses independent transcriptional programs in neurons.

### Pathological roles of HIF-mediated ferroptosis in ischemic stroke

Ferroptosis is a significant contributor to the pathogenesis of neurological diseases (Dixon & Stockwell, 2014; Li et al., 2017a). In ischemic stroke, hypoxia and oxidative stress induce ferroptosis in neural cells, exacerbating brain injury and worsening clinical outcomes (Li et al., 2017a; Zhang et al., 2018). The dual role of HIF-1α reflects its context-dependent regulation, wherein the duration and intensity of hypoxia determine its pro-survival or pro-death outcomes.

Table 2 summarizes the pathological mechanisms and clinical correlations of HIF-mediated ferroptosis in ischemic stroke. In the pathological context of ischemic stroke, the “double-edged sword” nature of HIF-mediated ferroptosis becomes fully apparent.

**Table 2:** Mechanisms of HIF-mediated ferroptosis in ischemic stroke. Upward arrows indicate an increase, and downward arrows indicate a decrease.

**HIF isoform**	**Mechanism/effect**	**Molecular pathways/targets**	**Pathological outcome**	**Key references**
HIF-1α	Promotes ferroptosis (Pro-death)	↑PTGS2, AIFM2, NCOA4; ↓Slc7a11, FSP1; ↑free Fe^2+^*via* modulation of iron metabolism genes	Enhances oxidative stress, worsens neuronal survival, enlarges infarct	Amidfar, Garcez & Kim, 2023; Li et al., 2021; Wang et al., 2025; Yang et al., 2014; Zhang et al., 2021b
		Upregulates ROS-producing enzymes (*e.g.*, NOX4)	Promotes ferroptotic injury under hypoxia	Chen et al., 2021a; Zhao et al., 2022
	Neuroprotective Role (Pro-survival)	Transient ↓ACSL4; ↑EPO, VEGF; Modulation of adiponectin signaling	Alleviates ferroptosis, promotes angiogenesis, reduces microglial damage	Cui et al., 2021; Liu et al., 2023; Zhang et al., 2021b
	Clinical correlation	Elevated serum HIF-1α correlates with neurological deficit scores	Indicator of stroke severity	Amalia et al., 2020; Liao et al., 2025; Zhu et al., 2024b
HIF-3α	Regulates Ferroptosis *via* Genetic and Mitochondrial Mechanisms	↑GPX4 axis; mitochondrial ROS modulation; histone deacetylase 1 recruitment	May protect against or modulate cerebral infarction	Ando et al., 2013; Jiang et al., 2024; Li et al., 2019
	Gene Variant Linked to Stroke Susceptibility	rs3826795 SNP in HIF-3α intron	Genetic predisposition to ischemic stroke	Gu et al., 2021
	Tissue Distribution and Hypoxic Induction	↑HIF-3α mRNA in cortex, hippocampus, lungs, heart	Highlights its systemic and CNS-specific hypoxia response	Duan, 2016; Zhang et al., 2014

### The “double-edged sword” effect of HIF-1α

Under hypoxic/ischemic conditions, HIF-1α activation directly contributes to ferroptosis in neural cells. Mechanistically, HIF-1α promotes the initiation and execution of ferroptosis by coordinately upregulating key pro-ferroptotic mediators such as prostaglandin-endoperoxide synthase 2 (PTGS2), and nuclear receptor coactivator 4 (NCOA4), which enhance lipid peroxidation and ferritinophagy, respectively (Gao et al., 2016; Li et al., 2021; Zhang et al., 2021b), while concurrently suppressing ferroptosis inhibitors including solute carrier family 7 member 11 (SLC7a11), FSP1; also known as apoptosis-inducing factor mitochondria-associated 2 (AIFM2), and GPX4, thereby diminishing antioxidant capacity and enhancing lipid peroxidation (Amidfar, Garcez & Kim, 2023; Bersuker et al., 2019). In experimental ischemic stroke models, increased HIF-1α activity correlates positively with neuronal ferroptosis, oxidative stress, and infarct severity, whereas pharmacological inhibition reduces lipid ROS accumulation and neuronal injury (Jurcau & Simion, 2021; Karuppagounder & Ratan, 2012; Lewén, Matz & Chan, 2000; (Li et al., 2017a; Zhang et al., 2018; Zhao et al., 2022). Clinical observations further demonstrate that circulating HIF-1α levels correlate with neurological deficit severity in stroke patients (Hilkens et al., 2024).

Despite these pro-ferroptotic effects, accumulating evidence indicates that HIF-1α also exerts context-dependent neuroprotective functions. During early ischemia-reperfusion, moderate and transient HIF-1α activation has been shown to suppress ACSL4 expression, thereby alleviating ferroptotic susceptibility and improving functional recovery (Cui et al., 2021; Liu et al., 2023). In the subacute phase, HIF-1α induces EPO and VEGF, promoting angiogenesis, neurovascular remodeling, and suppression of microglial hyperactivation within the ischemic penumbra (Amin et al., 2024; Liao et al., 2025; Wang, Li & Liu, 2023). HIF-1α has also been implicated in adiponectin-mediated antioxidant and anti-apoptotic pathways that protect against secondary brain injury (Zhang et al., 2021b).

Collectively, these findings support a dual and temporally dynamic role of HIF-1α in ischemic stroke. Under severe and sustained hypoxia, particularly within the ischemic core, HIF-1α signaling predominantly amplifies iron-dependent lipid peroxidation and ferroptotic neuronal death. Conversely, in the penumbral region or during early reperfusion, controlled activation facilitates metabolic adaptation and tissue repair. The apparent discrepancies across studies likely reflect differences in injury timing (acute ischemia *versus* reperfusion), oxygen gradients (core *versus* penumbra), cellular context (neurons *versus* glia), and experimental systems (*in vitro* hypoxia *versus in vivo* middle cerebral artery occlusion (MCAO) models) (Karuppagounder & Ratan, 2012; Lewén, Matz & Chan, 2000). Thus, HIF-1α functions not as a strictly pro-death or pro-survival factor, but as a dynamic regulator whose net impact on ferroptosis is determined by microenvironmental and temporal variables.

### HIF-3α regulation of ischemic stroke

In contrast to HIF-1α, evidence supporting a direct ferroptosis-specific role for HIF-3α in ischemic stroke remains limited and largely indirect. Hypoxic exposure has been shown to increase HIF-3α mRNA expression in the cortex and hippocampus of adult rats (Heidbreder et al., 2003). In addition, genetic association analyses have identified correlations between HIF-3α polymorphisms, particularly the intronic rs3826795 variant and increased susceptibility to ischemic stroke (Gu et al., 2021). While these findings suggest potential involvement in hypoxia-responsive signaling and stroke risk, they do not establish a causal link between HIF-3α and ferroptotic regulation in cerebral ischemia.

Mechanistic insights derived primarily from non-cerebral disease models propose that HIF-3α may interact with mitochondrial and redox regulatory pathways. For example, emerging evidence indicates that HIF-3α may influence antioxidant systems, including GPX4-associated pathways (Jiang et al., 2024). Moreover, its localization to the mitochondrial outer membrane under both normoxic and hypoxic conditions suggests a potential role in oxidative stress modulation and mitochondrial homeostasis (Duan, 2016). Given the central importance of mitochondrial dysfunction and lipid peroxidation in ferroptosis, these observations provide a plausible but currently unverified mechanistic link between HIF-3α and ferroptotic processes.

However, direct experimental evidence demonstrating that HIF-3α modulates ferroptosis in *in vivo* ischemic stroke models remains insufficient. To date, most available data rely on transcriptional profiling or genetic association studies rather than functional ferroptosis-specific assays. Furthermore, the existence of multiple HIF-3α splice variants, which exhibit distinct and sometimes opposing transcriptional activities, complicates mechanistic interpretation and limits definitive conclusions. Accordingly, the role of HIF-3α in ischemic stroke-associated ferroptosis should be regarded as preliminary and hypothesis generating rather than established. Future investigations employing isoform specific genetic manipulation, conditional knockout models, and validated ferroptosis biomarkers will be necessary to clarify its functional contribution.

Taken together, current evidence suggests that while HIF-1α has a well characterized dual and temporally dynamic role in ferroptotic regulation during ischemic stroke, the contribution of HIF-3α remains incompletely defined. This distinction is important for therapeutic considerations, as premature targeting of HIF-3α without mechanistic validation may lead to unpredictable outcomes.

### Potential therapeutic applications of HIF-regulated ferroptosis

Ferroptosis has emerged as a potential therapeutic target across various diseases, including cancer, neurodegeneration, and ischemic injury (Bao et al., 2023; Li et al., 2020b; Long et al., 2023; Motooka & Toyokuni, 2023; Peeples & Genaro-Mattos, 2022). In ischemic stroke, hypoxia-induced stabilization of HIFs, particularly HIF-1α, intersects with oxidative stress, iron dysregulation, lipid peroxidation, mitochondrial impairment, and inflammatory cascades, thereby positioning HIF-regulated ferroptosis at the centre of ischemic pathophysiology (Ratan, 2020; Wang et al., 2021; Zhang et al., 2020).

Given the pathological involvement of HIF-1α and the emerging but less defined contribution of HIF-3α in ischemic stroke, therapeutic modulation of HIF-regulated ferroptosis represents a conceptually attractive yet mechanistically complex strategy.

As discussed earlier, HIF-1α orchestrates ferroptotic signaling through coordinated regulation of iron metabolism, lipid peroxidation, glutathione depletion, and mitochondrial function. However, the dual and context-dependent roles of HIF-mediated ferroptosis necessitate precisely calibrated therapeutic approaches rather than uniform activation or inhibition. Sustained and excessive HIF-1α activation under prolonged hypoxia promotes iron accumulation, lipid ROS generation, and neuronal ferroptosis, thereby exacerbating infarct progression. In contrast, controlled or transient HIF-1α activation during early ischemia-reperfusion or within the ischemic penumbra may enhance metabolic adaptation, angiogenesis, and neurovascular remodeling, ultimately supporting tissue recovery (Amin et al., 2024; Cui et al., 2021; Liu et al., 2023; Wang, Li & Liu, 2023).

These temporally dynamic effects underscore the importance of spatial and stage-specific therapeutic modulation (Amin et al., 2024; Wang, Li & Liu, 2023). Strategies aimed at fine tuning HIF-regulated ferroptotic signaling such as selective inhibition of iron loading pathways and ferritinophagy (Gao et al., 2016; Li et al., 2021), restoration of antioxidant systems including SLC7A11, FSP1, and GPX4 (Amidfar, Garcez & Kim, 2023; Bersuker et al., 2019), or modulation of downstream lipid peroxidation cascades (Li et al., 2017a; Zhang et al., 2018) may offer greater clinical precision than global HIF suppression, which could impair adaptive angiogenic and metabolic responses (Amin et al., 2024; Wang, Li & Liu, 2023).

Increasing experimental evidence indicates that pharmacological agents and natural compounds can modulate HIF-associated pathways and downstream redox networks, thereby influencing ferroptotic susceptibility in preclinical ischemic models (Wang et al., 2021; Zhang et al., 2020). Nevertheless, current data remain largely derived from *in vitro* hypoxia systems or rodent MCAO models, and translation to human stroke therapy is limited by differences in timing of intervention, blood–brain barrier permeability, systemic iron metabolism, and patient heterogeneity.

Therefore, while targeting HIF-regulated ferroptosis is mechanistically grounded and translationally promising, future studies must define optimal therapeutic windows, clarify isoform-specific effects, and incorporate clinically relevant outcome measures before routine clinical application can be considered.

### Targeting HIF-regulated ferroptosis-related genes

As discussed above, HIF-1α regulates multiple ferroptosis-related effectors, including PTGS2, AIFM2 (FSP1), and NCOA4 (Yang et al., 2014; Zhang et al., 2021b). Pharmacological targeting of these downstream mediators may attenuate ferroptotic neuronal death. In addition, HIF-1α influences the expression of ferroptosis suppressors such as Slc7a11 and GPX4-related antioxidant systems (Bersuker et al., 2019; Gao et al., 2016), suggesting that restoration of redox buffering capacity may counteract hypoxia-driven ferroptosis.

Experimental studies further demonstrate that modulation of antioxidant pathways converging on glutathione metabolism and lipid peroxidation mitigates ischemia-reperfusion injury (Wang et al., 2021). Because ferroptosis is characterized by glutathione depletion and excessive lipid ROS accumulation, targeting these HIF-regulated redox nodes provides a rational therapeutic approach.

### Modulators of HIF-1α activity: experimental evidence in ischemic stroke

Direct modulation of HIF-1α activity represents another promising therapeutic strategy in ischemic stroke (Ratan, 2020; Vatte & Ugale, 2023; Wang et al., 2021). However, the dual and context-dependent nature of HIF-1α exerting both pro-ferroptotic and adaptive neuroprotective effects depending on injury stage necessitates careful therapeutic calibration (Amin et al., 2024; Karuppagounder & Ratan, 2012; Wang, Li & Liu, 2023). Several compounds have demonstrated neuroprotective efficacy in experimental ischemic models through modulation of HIF-associated pathways and downstream redox networks (Li et al., 2017b; Zhang et al., 2018; Zhang et al., 2020; Zhao et al., 2022).

Berberine (BBR) regulates hypoxia signaling *via* activation of the sphingosine-1-phosphate (S1P)/HIF-1 pathway, contributing to neuronal protection under hypoxia/ischemia (Zhang et al., 2016a). In ischemic models, BBR attenuates Ca^2+^ overload, suppresses apoptosis, and promotes vascular remodeling through HIF-related mechanisms (Liu, Ren & Ma, 2018; Nadjafi, Ebrahimi & Rahbar-Roshandel, 2014a; Nadjafi, Ebrahimi & Rahbar-Roshandel, 2014b). Given the close relationship between HIF signaling, iron metabolism, and oxidative stress, these findings suggest that BBR may indirectly influence ferroptotic vulnerability.

Ginkgolides upregulate HIF-1α protein expression in hypoxic neuronal models while reducing LDH release and improving neuronal viability (Zhu et al., 2007a; Zhu et al., 2007b). Additionally, ginkgolides enhance antioxidant defences *via* activation of the nuclear factor erythroid 2-related factor 2 (Nrf2) pathway (Li et al., 2019; Ye et al., 2022). Since Nrf2-driven transcription strengthens glutathione synthesis and limits lipid peroxidation (Li et al., 2024b; Wang et al., 2021), this mechanism may counteract ferroptosis under ischemic conditions.

Curcumin exerts neuroprotection in transient MCAO models by activating the PI3K/Akt/Nrf2 axis, reducing inflammation, suppressing excessive autophagy, and enhancing antioxidant capacity (Wang et al., 2021). Because PI3K/Akt signaling contributes to HIF-1α stabilization and metabolic adaptation, curcumin may indirectly modulate ferroptotic regulation through coordinated redox control.

Moreover, inhibition of NADPH oxidase (NOX)-mediated ROS production reduces oxidative injury in ischemic stroke models (Wang et al., 2021). As ROS accumulation contributes to both HIF stabilization and lipid peroxidation, NOX inhibition may represent an additional mechanism through which compounds attenuate HIF-associated ferroptotic signaling.

Generally, these findings support the concept that pharmacological modulation of hypoxia-responsive signaling networks significantly alters oxidative stress and iron-dependent lipid damage in experimental stroke models.

### Therapeutic implications of HIF-3α

Beyond HIF-1α, HIF-3α has recently gained attention as a potential regulator of oxidative stress and ferroptosis-related pathways. Activation of the HIF-3α-GPX4 axis has demonstrated protective effects in hypoxia-associated disease contexts (Jiang et al., 2024), suggesting possible parallels in ischemic stroke. Hypoxia-enhanced mitochondrial outer membrane localization of HIF-3α provides additional mechanistic insights into its role in regulating mitochondrial redox homeostasis (Duan, 2016).

Although research on HIF-3α remains limited compared with HIF-1α, its isoform-specific functions may offer opportunities for selective modulation of ferroptosis without broadly disrupting adaptive hypoxic responses.

### Phytotherapy targeting broader signaling networks intersecting with HIF and ferroptosis

In addition to direct modulation of HIFs, growing evidence highlights the therapeutic potential of phytochemicals that target upstream signaling networks, including phosphoinositide 3-kinase/protein kinase B (PI3K/Akt), Nrf2, NF-κB, and AMP-activated protein kinase (AMPK) which converge on hypoxia signaling and ferroptosis regulation. Rather than acting on a single molecular node, many natural compounds exert pleiotropic effects that influence HIF stability, redox homeostasis, inflammatory signaling, and lipid peroxidation pathways.

Natural products such as quercetin, berberine, and curcumin have been shown to modulate PI3K/Akt signaling in ischemic models (Xu et al., 2023; Zhang et al., 2016b). Because PI3K/Akt activity contributes to HIF-1α stabilization and regulates cellular survival and metabolic adaptation, phytotherapeutic modulation of this pathway may indirectly alter ferroptotic susceptibility under hypoxic stress. This upstream regulatory approach may provide a more physiologically balanced alternative to complete HIF inhibition.

Activation of the Nrf2 antioxidant axis represents another critical intersection between phytotherapy and ferroptosis. Nrf2 induces expression of HO-1, glutamate-cysteine ligase (GCL), and related antioxidant enzymes that enhance glutathione synthesis and attenuate lipid peroxidation (Wang et al., 2021). Ginkgolides and curcumin have been reported to enhance Nrf2 signaling in ischemic contexts (Wang et al., 2021; Ye et al., 2022). Given that ferroptosis is characterized by iron-dependent lipid peroxidation and glutathione depletion, reinforcement of Nrf2-mediated antioxidant defences may counteract HIF-associated ferroptotic cascades.

Comprehensive analyses of natural products in hypoxia-induced neurological injury further emphasize their multi-target properties, including anti-inflammatory, anti-oxidative, anti-apoptotic, and HIF-modulatory effects (Zhang et al., 2026). Collectively, these findings position phytotherapy as a systems-level regulator of interconnected hypoxia-responsive networks rather than a single-pathway intervention.

From a translational standpoint, preclinical studies demonstrate that targeting HIF-1α or its downstream ferroptotic mediators reduces oxidative damage, lipid peroxidation, and infarct volume in experimental stroke models (Li et al., 2017b; Zhang et al., 2018; Zhao et al., 2022). Similarly, pharmacological modulation of intersecting pathways such as PI3K/Akt and Nrf2-dependent antioxidant systems confers neuroprotection in ischemia reperfusion paradigms (Wang et al., 2021; Zhang et al., 2020). However, clinical translation remains constrained by several factors, including the temporal complexity of HIF signaling where phase-specific intervention is essential to preserve endogenous repair processes (Amin et al., 2024; Wang, Li & Liu, 2023), as well as reliance on rodent MCAO models that may not fully recapitulate human stroke heterogeneity (Karuppagounder & Ratan, 2012). In addition, reliable clinical biomarkers reflecting ferroptosis activity remain underdeveloped, although circulating HIF-1α levels have been associated with neurological deficit severity (Hilkens et al., 2024). Pharmacokinetic limitations, including blood–brain barrier penetration and potential systemic off target effects, further complicate therapeutic implementation.

Therefore, while phytotherapy targeting HIF-intersecting ferroptotic networks holds translational promise, future progress will require improved bioavailability strategies, biomarker-guided patient stratification, and validation in clinically relevant models.

### Challenges and future perspectives in targeting HIF-mediated ferroptosis

Despite compelling mechanistic and preclinical evidence, translating HIF-regulated ferroptosis modulation into clinically effective therapies for ischemic stroke remains challenging. Experimental studies demonstrate that targeting HIF-1α or its downstream ferroptotic mediators reduces oxidative damage, lipid peroxidation, and infarct volume in rodent MCAO models (Li et al., 2017b; Zhang et al., 2018; Zhao et al., 2022). Additionally, pharmacological modulation of intersecting pathways including PI3K/Akt and Nrf2-dependent antioxidant systems has shown neuroprotective effects in ischemia-reperfusion paradigms (Wang et al., 2021; Zhang et al., 2020). Clinical observations further indicate that circulating HIF-1α levels correlate positively with neurological deficit severity, suggesting potential biomarker relevance (Hilkens et al., 2024).

However, several translational barriers must be addressed. First, the dual and temporally dynamic roles of HIF-1α necessitate precise spatial and phase-specific modulation to avoid suppressing endogenous protective responses, such as angiogenesis and metabolic adaptation during recovery stages (Amin et al., 2024; Wang, Li & Liu, 2023). Uniform or prolonged inhibition may therefore produce unintended adverse effects.

Second, most mechanistic insights derive from *in vitro* hypoxia systems or rodent MCAO models, which may not fully capture the biological heterogeneity, comorbidities, and therapeutic timing constraints characteristic of human ischemic stroke (Karuppagounder & Ratan, 2012). Bridging this translational gap will require validation in large animal models and incorporation of clinically relevant endpoints.

Third, reliable biomarkers that specifically reflect ferroptosis activity in patients remain underdeveloped. Future clinical studies should incorporate ferroptosis-related endpoints such as ACSL4 expression, GPX4 activity, lipid ROS accumulation, and iron dysregulation to determine whether HIF-modulating interventions exert bona fide anti-ferroptotic effects *in vivo*.

Fourth, pharmacokinetic limitations including restricted blood–brain barrier penetration, limited bioavailability of certain phytochemicals, and potential systemic off-target effects pose substantial obstacles to therapeutic implementation (Zhang et al., 2026). Advances in nanodelivery platforms, structural optimization of small-molecule modulators, and targeted formulation strategies may enhance brain-specific delivery and improve therapeutic indices.

Overall, these considerations indicate that successful clinical translation will require biomarker-guided patient stratification, precise temporal targeting aligned with ischemia-reperfusion dynamics, and combinatorial strategies that recalibrate rather than indiscriminately suppress hypoxia-adaptive signaling networks. Therapeutic targeting of HIF-mediated ferroptosis should therefore be conceptualized not merely as inhibition of cell death, but as stage-specific restoration of redox and iron homeostasis during ischemic stroke progression.

## Conclusions

The HIF family exerts a dual role in ischemic stroke, driving both tissue injury and cytoprotection through its multifaceted regulation of ferroptosis. This review has detailed the mechanisms by which HIF-1α and HIF-3α modulate ferroptosis and explored their potential applications in neuroprotection and stroke therapy. To date, research on HIF-1α in ischemic stroke has yielded substantial findings, revealing its capacity to protect neural cells from hypoxia-induced damage by activating or inhibiting diverse ferroptosis-related molecular pathways. In contrast, the study of HIF-3α remains in its infancy. Future research should prioritize the development of isoform-specific HIF modulators and elucidate the functional hierarchy of HIF-3α splice variants in the brain. Overcoming the challenges of therapeutic timing, specificity, and delivery will be crucial for harnessing the full therapeutic potential of the HIF-ferroptosis axis. Notably, this review provides the first comprehensive integration of the distinct regulatory networks of HIF-1α and HIF-3α in ischemic stroke-associated ferroptosis, laying the groundwork for future endeavors aimed at mitigating stroke-induced neurological deficits.

## Additional Information and Declarations

### Data Availability

The following information was supplied regarding data availability: This is a literature review.
